# Special Issue: Innovative Material Design and Nondestructive Testing Applications for Infrastructure Materials

**DOI:** 10.3390/ma18030611

**Published:** 2025-01-29

**Authors:** Honglei Chang, Feng Guo

**Affiliations:** School of Qilu Transportation, Shandong University, Jinan 250003, China; fengg@sdu.edu.cn

Construction materials play a vital role in the design, construction, and maintenance of transportation infrastructure, significantly impacting its safety, durability, and stability. The rapid development of global urbanization and the increasing demand for transportation intensified challenges, including aging materials, environmental degradation, and the impacts of climate change. To address these issues, the development of innovative materials and advanced nondestructive testing techniques is essential. Researchers are actively developing new construction materials, enhancing the performance of existing ones, and advancing cutting-edge nondestructive testing methods to meet the evolving requirements of modern, increasingly complex transportation infrastructure.

This Special Issue, titled “Innovative Material Design and Nondestructive Testing Applications for Infrastructure Materials,” showcases cutting-edge research and technological advancements in the field of infrastructure materials. Featuring ten high-quality research articles developed over nearly a year, this collection covers a diverse range of topics in innovative material design and nondestructive testing techniques. Contributions from more than 40 authors representing various organizations provide comprehensive insights and perspectives on this rapidly evolving and critical area of study.

Dong et al. [1] investigated the impact of coarse particles, such as sand and walnut shells, on filter cake formation and air tightness in high-permeability formations. Their findings revealed that walnut shells were more effective than sand in improving the airtightness of filter cakes, and they recommended incorporating walnut shells at a rate of 30–40 g/L for formations with permeability coefficients greater than 1.0 × 10^−3^ m/s. Nering and Nering [2] assessed vibration isolation materials for reducing urban noise and vibrations, employing a single-degree-of-freedom system and image-processing techniques. Their study demonstrated that the image-processing method reliably predicted the materials’ dynamic stiffness and damping properties, revealing strong correlations between indentation and dynamic stiffness, as well as rebound and damping. Haruna et al. [3] studied the bonding behavior of NSC-UHPFRC composites under impact loading. Their findings revealed that surface treatment methods significantly influenced bond strength, and the XGBoost model accurately predicted impact strength. Liu et al. [4] explored the effects of freeze–thaw cycling and cyclic loading on the damage evolution of sandstone. The results showed that freeze–thaw cycling increased sandstone’s total porosity and microporosity in a linear fashion. Using the loading–unloading response ratio and the strain equivalence principle, a damage model was developed for fractured rock under freeze–thaw–fatigue coupling. Peng et al. [5] examined the bond degradation between polyurea coatings and concrete substrates using accelerated aging experiments. They found that bond strength declined over time and developed an aging model to predict the coating’s service life.

Fu et al. [6] analyzed sandstone damage evolution under high strain rates using a split Hopkinson pressure bar. They observed that internal stress inhomogeneity remained within 5% across strain rates of 931–2250 s^−1^, highlighting the influence of deformation stages and strain rates on material behavior. Behnia and Lukaszewski [7] proposed a novel method combining acoustic emission, machine learning, and digital image techniques to analyze spiral crack patterns in soft binders. The study found strong correlations between helical crack energy and properties such as fracture energy and embrittlement temperature. He et al. [8] investigated the axial compressive properties of recycled aggregate concrete reinforced with ultra-high toughness cementitious composites. Steel fibers significantly enhanced the mechanical properties of the structures, with the study recommending their use for applications requiring higher mechanical strength. Li et al. [9] developed a pavement icing detection system based on a piezoelectric sensor, integrated with a BP neural network for early warning. The system achieved a prediction accuracy exceeding 90%, offering an effective approach for winter road safety monitoring. Ji et al. [10] analyzed the permanent deformation of asphalt pavement-bearing layers and proposed a control criterion based on dynamic modulus and stability. This approach aims to improve the deformation resistance of bearing layers, thereby extending the pavement’s service life.

As a multidisciplinary and expansive topic, “Innovative Material Design and Nondestructive Testing Applications for Infrastructure Materials” cannot be comprehensively explored within the scope of a single Special Issue. Nevertheless, the papers included in this issue provide valuable insights into some of the critical challenges in the field. They highlight the diverse applications of innovative material design and nondestructive testing techniques in infrastructure. It is our hope that these findings will serve as a foundation for further research and inspire continued advancements, contributing to the progress of infrastructure materials science and engineering.

## References

[1] Dong Q., Liu T., Wang Y., Liu S., Wen L. (2024). Study on the Influence of Walnut Shell Coarse Particles on the Slurry Permeation and the Air Tightness of Filter Cake. Materials.

[2] Nering K., Nering K. (2024). Alternative Method for Determination of Vibroacoustic Material Parameters for Building Applications. Materials.

[3] Haruna S.I., Ibrahim Y.E., Hassan I.H., Al-shawafi A., Zhu H. (2024). Bond Strength Assessment of Normal Strength Concrete–Ultra-High-Performance Fiber Reinforced Concrete Using Repeated Drop-Weight Impact Test: Experimental and Machine Learning Technique. Materials.

[4] Liu T., Cai W., Sheng Y., Huang J. (2024). Experimental Study on the Microfabrication and Mechanical Properties of Freeze–Thaw Fractured Sandstone under Cyclic Loading and Unloading Effects. Materials.

[5] Peng C., Ren J., Wang Y. (2024). Degradation Behavior and Lifetime Prediction of Polyurea Anti-Seepage Coating for Concrete Lining in Water Conveyance Tunnels. Materials.

[6] Fu Y., Chen S., Zhao P., Ye X. (2023). The Mechanism of Deformation Compatibility of TA2/Q345 Laminated Metal in Dynamic Testing with Split-Hopkinson Pressure Bar. Materials.

[7] Behnia B., Lukaszewski M. (2023). Novel Approach in Fracture Characterization of Soft Adhesive Materials Using Spiral Cracking Patterns. Materials.

[8] He L., Peng S., Jia Y.-S., Yao Y.-K., Huang X.-W. (2023). Testing and Analysis of Ultra-High Toughness Cementitious Composite-Confined Recycled Aggregate Concrete under Axial Compression Loading. Materials.

[9] Li J., Ma H., Shi W., Tan Y., Xu H., Zheng B., Liu J. (2023). Nondestructive Detection and Early Warning of Pavement Surface Icing Based on Meteorological Information. Materials.

[10] Ji W., Meng Y., Shang Y., Zhou X., Xu H. (2023). Investigation of the Relationship between Permanent Deformation and Dynamic Modulus Performance for Bearing-Layer Asphalt Mixture. Materials.

